# Comparison of the gastrointestinal bacterial microbiota between dairy cows with and without mastitis

**DOI:** 10.3389/fmicb.2024.1332497

**Published:** 2024-03-22

**Authors:** Chunyan Guo, Jingjing Liu, Yong Wei, Wen Du, Shengli Li

**Affiliations:** ^1^State Key Laboratory of Animal Nutrition, College of Animal Science and Technology, China Agricultural University, Beijing, China; ^2^Jinzhong Vocational and Technical College, Jinzhong, China; ^3^Xinjiang Agricultural University, Urumuqi, China

**Keywords:** mastitis, cows, microbiota, rumen, fece

## Abstract

Mastitis causes significant losses in the global dairy industry, and the health of animals has been linked to their intestinal microbiota. To better understand the relationship between gastrointestinal microbiota and mastitis in dairy cows, we collected blood, rumen fluid, and fecal samples from 23 dairy cows, including 13 cows with mastitis and 10 healthy cows. Using ELISA kit and high-throughput sequencing, we found that cows with mastitis had higher concentrations of TNF-α, IL-1, and LPS than healthy cows (*p* < 0.05), but no significant differences in microbiota abundance or diversity (*p* > 0.05). Principal coordinate analysis (PCOA) revealed significant differences in rumen microbial structure between the two groups (*p* < 0.05), with *Moryella* as the signature for rumen in cows with mastitis. In contrast, fecal microbial structure showed no significant differences (*p* > 0.05), with *Aeriscardovia*, *Lactococcus*, and *Bacillus* as the signature for feces in healthy cows. Furthermore, the results showed distinct microbial interaction patterns in the rumen and feces of cows with mastitis compared to healthy cows. Additionally, we observed correlations between the microbiota in both the rumen and feces of cows and blood inflammatory indicators. Our study sheds new light on the prevention of mastitis in dairy cows by highlighting the relationship between gastrointestinal microbiota and mastitis.

## 1 Introduction

Mastitis is a common challenge in the dairy industry (Krishnamoorthy et al., 2021). It can cause serious economic losses and costs to the farm in terms of decreased milk yield, changed milk composition, decreased milk quality, expenditure on drugs, veterinary fees, increased labor, discarded milk, costs incurred on replacement heifers, reduced slaughter value, idle production factors, and lost future income that results from culling (Jamali et al., 2018; Dolecheck et al., 2019; Sah et al., 2020). In dairy production, the somatic cell counts (SCCs) in milk are the primary criterion for determining whether cows are suffering from mastitis. 200,000 cells/mL ≤ an SCC ≤ 500,000 cells/mL is defined as subclinical mastitis, and 500,000 cells/mL < an SCC is defined as clinical mastitis (Sharma et al., 2011). Additionally, cow with mastitis can result in noticeable abnormalities in milk, such as changes in color and the presence of fibrin clots. As the inflammation level increases, changes in the udder, including swelling, heat, pain, and redness, may also become apparent. Generally, mastitis in cows is caused by pathogenic bacteria invading the breast tissue (Smith et al., 1985; Mungube et al., 2004). However, in some cases, antibiotic intervention is not effective, leading to chronic inflammatory processes, breast fibrosis, and atrophy (Pedersen et al., 2021). Hence, there is a need to explore the newly underlying mechanisms of mastitis.

Recent research has focused on the role of gut microbiota, a complex ecosystem that has been associated with health in both humans and animals (Fan and Pedersen, 2021; Nathan et al., 2021). Many studies have shown that gut microbiota can participate in the regulation of human nutrient absorption and immune functions as well as the occurrence of human diseases (Gill et al., 2021; Klag and Round, 2021; Matson et al., 2021). Unlike monogastric animals, ruminants have a unique rumen where the microbiota, along with the intestinal microbiota, collaboratively play a role in maintaining the life activities of ruminants. Recent studies have also found that rumen and hindgut microbiota of cows contribute significantly to the health, affecting conditions such as subacute ruminal acidosis, left-sided displacement of the abomasum, and diet-induced milk fat depression (Mao et al., 2013; Oikonomou et al., 2013; Song et al., 2016; Pitta et al., 2018). Interestingly, Wang et al. (2021, 2022) also found structural differences in ruminal and fecal microbiota between mastitis and healthy cows. Meanwhile, Zhao et al. (2022) and Ma et al. (2018) revealed that transplanting ruminal fluid and feces from cows with mastitis to germ-free mice causes the mice to develop mastitis, whereas mice transplanted with ruminal fluid and feces from healthy cows do not develop mastitis. These findings suggest that gastrointestinal microbiota is also one of the major contributors to mastitis in cows. Furthermore, studies have shown that certain cytokines, such as interleukin-1 (IL-1), tumor necrosis factor-alpha (TNF-α), and interleukin-6 (IL-6), are significantly elevated in cows with mastitis, reflecting the immune response to udder infection (Zhu et al., 2007). These cytokine profiles could be linked to alterations in the rumen microbiota, suggesting a complex interaction between microbial dysbiosis and immune responses in mastitis pathogenesis. In summary, the disruption of this microbiota balance might result in an increased prevalence of pathogenic bacteria and a decrease in beneficial bacteria, potentially causing metabolic disturbances in the rumen or intestines. Consequently, this disruption can impair immune responses and increase susceptibility to mastitis (Bronzo et al., 2020; Wang et al., 2021; Hu et al., 2022). Understanding these changes is crucial, as they could offer new insights into preventive and therapeutic strategies against mastitis. However, in these studies, consistent key bacteria have not been identified, possibly due to inconsistencies in samples, lactation stages, and diets. Currently, there are no reports on the differences in the microbial composition between the rumen and the hindgut (feces) of cows with mastitis within the same case, and it has not been reported which of the microbiota, the rumen or the hindgut, has a greater impact on mastitis.

Therefore, this study investigates the microbial composition of the rumen and feces between 13 Holstein cows with clinical mastitis and 10 healthy Holstein cows at the same lactation stage. We hypothesize that under the same feeding management conditions, there are significant differences in the rumen and fecal microbiota between healthy and mastitis cows. Furthermore, due to the higher diversity of microbiota in the rumen, we assume that the rumen microbiota has a more significant impact on the development of mastitis compared to the fecal microbiota. Our study seeks to provide insights into the pathogenesis of mastitis in dairy cows.

## 2 Materials and methods

### 2.1 Animal, management, and clinical diagnosis

This study was conducted at the Zhongdi Dairy Research Center in Beijing, China. In the experiment, all cows were housed in the same barn and had access to a total mixed ration (TMR) with a forage-to-concentrate ratio of 60:40 and water *ad libitum*. The TMR was added three times a day (0,700, 1,430, and 2,200), and its formulation complied with NRC (2001) requirements. The composition and nutritional information of the TMR are shown in Table 1.

**Table 1:** Ingredients and chemical composition of the total mixed ration.

Item	Content
Ingredients	(% of feeding basis)
Corn silage	45.26
Alfalfa	13.76
Corn	1.40
Soybean meal	8.36
Extrusion Soybean	1.50
Flaked corn	14.64
Soybean hull	8.25
Cotton seed	2.80
Premix^a^	4.03
Total	100
Nutrient levels^b^	(% dry matter)
NEL/(MJ/kg)	7.06
CP	16.81
NDF	29.12
ADF	19.43
Ca	0.86
P	0.33

a One kilogram of complete diet (on dry matter basis) contained the following minerals and vitamin premix: Mn, 4,800 mg; Fe, 4,800 mg; Zn, 12,850 mg; Cu, 3,250 mg; I, 140 mg; Se, 150 mg; Co, 110 mg; Vitamin A, 1,000,000 IU; Vitamin D3, 280,000 IU; Vitamin E, 10,000 IU; niacin, 1,000 mg.

b NEL values were calculated using the net energy of lactation values of feedstuffs from NRC (2001); others were measured by laboratory analysis of the total mixed ration.

The study included a total of 23 Holstein cows, with 10 in the healthy group (H group) and 13 in the clinical mastitis group (M group). Clinical mastitis in cows was diagnosed by an experienced veterinarian. Initially, cows with recent decreases in milk production were selected using dairy management software (Valley Ag software, PA, United States). Subsequently, the veterinarian assessed the udders for clinical symptoms such as swelling, heat, and hardness using palpation in the milking parlor. Finally, milk samples from cows exhibiting clinical symptoms were collected and subjected to SCC testing using an instrument (Countess 3, Thermo Fisher, Waltham, United States), with 500,000 cells/mL set as the threshold for clinical mastitis, thus confirming cases of mastitis in the cows. Healthy cows were identified based on criteria such as normal milk production and udders free from abnormalities.

### 2.2 Sample collection

After identifying the sampled cows, we conducted the sampling on the following day. As described by our previous study, rumen fluid, feces, and blood samples from healthy cows were also collected before morning feeding. Rumen fluid samples were collected via sterilized esophageal tubing, fecal samples were obtained by inserting a hand (covered with a sterile glove) into the rectum, and blood samples were taken from the caudal root vein using 10 mL non-anticoagulant tubes. Blood samples were centrifuged at 1500 × g for 30 min at 4°C (Tiangen OSE-MP25, Beijing, China) to obtain serum, which was then stored in 1.5 mL centrifuge tubes. In the sampling period, no feces could be obtained from the rectum of one cow with mastitis. All samples were immediately snap-frozen in liquid nitrogen and stored at −80°C in a refrigerator until further experiments.

### 2.3 Serum cytokine and LPS detection

The concentrations of serum tumor necrosis factor-α (TNF-α, detection range: 0.1–1.6 pg./L), interleukin-8 (IL-8, detection range: 40–640 ng/L), IL-6 (detection range: 200–3,200 ng/L), IL-1 (detection range: 25–400 ng/L), and lipopolysaccharide (LPS, detection range: 0.01–100 EU/mL) were determined using respective ELISA kits (Nanjing Jian Cheng Bioengineering Institute, Nanjing, China).

### 2.4 DNA extraction, PCR amplification, and 16S rDNA sequencing

Rumen fluid and fecal samples, totaling 45 samples in all, underwent the extraction of microbial and fecal DNA. This extraction process followed the manufacturer’s guidelines and used the PowerSoil DNA Isolation Kit (MoBio Laboratories, Carlsbad, CA). The concentration and purity of the extracted DNA were assessed using a Nanodrop 2000 Spectrophotometer (Thermo Scientific, Waltham, MA, United States). The amplification of the V3–4 hypervariable region of the bacterial 16S rDNA gene was performed with the primers 338F (ACTCCTACGGGAGGCAGCAG) and 806R (GGAC-TACHVGGGTWTCTAAT). A 10-digit barcode sequence was added to the 5′ end of both the forward and reverse primers for each sample. The PCR was conducted in 25 μL reaction volumes on a Mastercycler Gradient (Eppendorf, Germany), comprising 12.5 μL of 2× Taq PCR MasterMix, 3 μL of BSA (2 ng/μL), 2 μL of each Primer (5 μM), 2 μL of template DNA, and 5.5 μL of ddH_2_O. The cycling parameters included an initial step at 95°C for 5 min, followed by 32 cycles of denaturation at 95°C for 45 s, annealing at 55°C for 50 s, and extension at 72°C for 45 s, with a final extension at 72°C for 10 min. To mitigate potential PCR biases at the reaction level, three PCR products per sample were pooled together.

### 2.5 Sequence analysis

The initial paired-end reads obtained from the original DNA fragments were merged using Flash version 1.20 (Magoč and Salzberg, 2011). Subsequently, each sample was segregated based on its unique barcode. After eliminating barcodes, primers, and splice variants, raw reads were acquired. These raw data were initially screened, and sequences were excluded from consideration if they fell below a length of 230 bp, had a quality score ≤20, contained ambiguous bases, or did not precisely match the primer sequences and barcode tags. Qualified reads were then separated using sample-specific barcode sequences and trimmed with Illumina Analysis Pipeline Version 2.6. Subsequently, the dataset underwent analysis using usearch (version 8.1). The sequences were grouped into operational taxonomic units (OTUs) at a 97% similarity level using the uparse method (Edgar, 2013), which allowed the generation of rarefaction curves. To classify the sequences into different taxonomic groups, the rdp Classifier tool (Wang et al., 2007) was employed, based on the SILVA ribosomal RNA gene database (Quast et al., 2013).

### 2.6 Statistical analyses

After the sample number of the lowest sequence was flattened, the species richness (observed species, chao1) and diversity (Shannon and Simpson index) were chosen for alpha-diversity and were calculated with OTUs data using QIIME (version v.1.8 http://qiime.org/scripts/alpha_rarefaction.html) (Caporaso et al., 2011; Fuentes et al., 2014); Intergroup alpha index variability was demonstrated by the Kruskal–Wallis test in R v.4.0.2. Principal coordinate analysis (PCoA) was performed based on Bray–Curtis dissimilarity matrices. The linear discriminant analysis effect size (LEfSe, LDA > 2) was used to identify dominant bacteria between two groups. The Spearman method was used in correlation analysis. Besides, based on the OTU data, we predicted the microbiota function in rumen and feces between the two groups using the PICRUSt2 software v2.4.1, and the results of predicted functions were analyzed and visualized through the STAMP software v2.1.3. The data, including milk yield, parity, age, days in milk, serum cytokine, and serum LPS concentration, were analyzed using one-way ANOVA in R v.4.0.2. All data are reported as means with a significance level set at 0.05.

## 3 Results

### 3.1 Apparent characteristics between healthy and mastitis cows

In this study, there are no significant differences in age, parity, and DIM of the cows between the two groups. To explore the inflammatory and immune status between healthy and mastitis cows, we next performed serum TNF-α, IL-8, IL-6, IL-1, and LPS tests and found that mastitis cows had higher (*p* < 0.05) serum TNF-α, IL-1, and LPS concentration (Table 2). These results indicated that mastitis cows had higher systemic inflammatory.

**Table 2:** Physical examination variables for cows between M and H group.

Items	M	H	*p*-value
Age (years)	5.1 ± 0.44	4.9 ± 0.81	0.527
DIM^a^ (days)	79 ± 2.10	80 ± 2.30	0.309
Parity	3.10 ± 0.64	2.90 ± 0.88	0.581
Milk yield (Kg/d)	29.4 ± 6.5	47.1 ± 8.3	<0.05
TNF-α (pg/L)	0.75 ± 0.16	0.63 ± 0.08	<0.05
IL-8 (ng/L)	141.0 ± 44.5	111.1 ± 29.34	0.104
IL-6 (ng/L)	509.3 ± 192.2	476.4 ± 199.8	0.747
IL-1 (ng/L)	117.7 ± 12.95	64.2 ± 20.74	<0.05
LPS (EU/mL)	0.12 ± 0.01	0.06 ± 0.01	<0.05

a DIM, days in milk; M, mastitis group; H, health group.

### 3.2 Diversity of gastrointestinal bacterial microbiota between healthy and mastitis cows

The amplicons from the V3–V4 region of 16S rDNA were sequenced for all the rumen and fecal samples. A total of 1,680,664 high-quality 16S rDNA gene sequences were obtained in the rumen samples with an average of 43,930 ± 4,871 per sample, and 1,932,935 high-quality 16S rDNA gene sequences were obtained in the fecal samples with an average of 40,992 ± 5,338 per sample. The Good’s coverage of all the samples is greater than 98%, indicating that the sequence coverage was deemed sufficient. In addition, we observed a total of 3,429 bacterial OTUs binned at 97% similarity, with an average 1,109 ± 98 OTUs in the rumen samples and 665 ± 169 OTUs in the fecal samples.

To understand the structure of gastrointestinal bacterial microbiota between the two groups, we used the observed species, Chao1, Shannon, and Simpson indexes based on the OTUs to evaluate the richness and diversity of microbiota. However, we found that there is no difference in the observed species, Chao1, Shannon, and Simpson indexes of rumen fluid or feces between the two groups (Table 3). The results showed that the richness and diversity of microbiota in rumen fluid or feces had similarity between heathy and mastitis cows.

**Table 3:** The α-diversity indices between the H and M groups at OTUs level.

Items	H	M	SEM	*p*-value
Rumen				
Observed species	1,083	1,039	25.5	0.46
Chao1	1,307	1,243	80.5	0.14
Shannon	5.49	5.42	0.21	0.58
Simpson	0.9882	0.9856	0.0014	0.42
Feces				
Observed species	718	692	33.1	0.79
Chao1	867	867.6	34.1	0.84
Shannon	4.59	4.52	0.14	0.84
Simpson	0.9689	0.9623	0.0042	0.51

M, mastitis group; H, health group.

**Figure 1:**
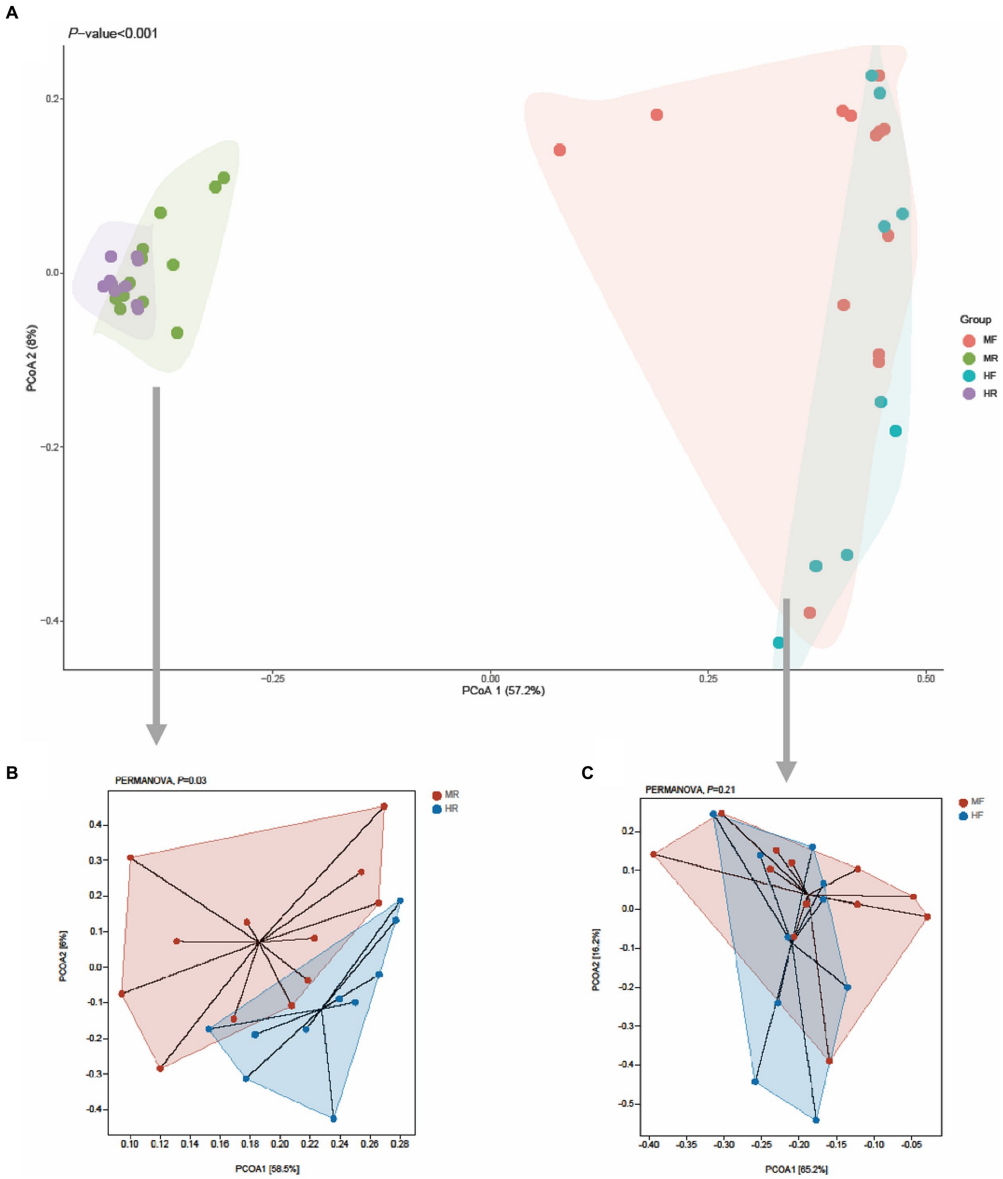
PCoA plots for the samples between the H and M groups based on the Bray–Curtis distance. **(A)** All samples, **(B)** rumen fluid, **(C)** feces; MR, ruminal samples in mastitis group; MF, fecal samples in mastitis group; HR, ruminal samples in health group; HF, fecal samples in health group.

Next, principal coordinates analysis (PCoA) based on Bray–Curtis was performed to determine whether the microbial community structure changed by mastitis (Figure 1). The results showed that clear separation of microbiota between rumen fluid and feces of cows at OTU level (PERMANOVA, *p* < 0.001, Figure 1A). Furtherly, we found that bacterial structure profiles of rumen fluid appeared significant segregation between two groups (PERMANOVA, *p* = 0.03, Figure 1B), while fecal bacterial microbiota had no difference (PERMANOVA, *p* = 0.21, Figure 1C). The results indicated that compared with the hindgut microbiota, ruminal microbiota may play a more important role in the development of mastitis in cows.

### 3.3 Taxononmic composition of gastrointestinal bacterial microbiota between healthy and mastitis cows

**Figure 2:**
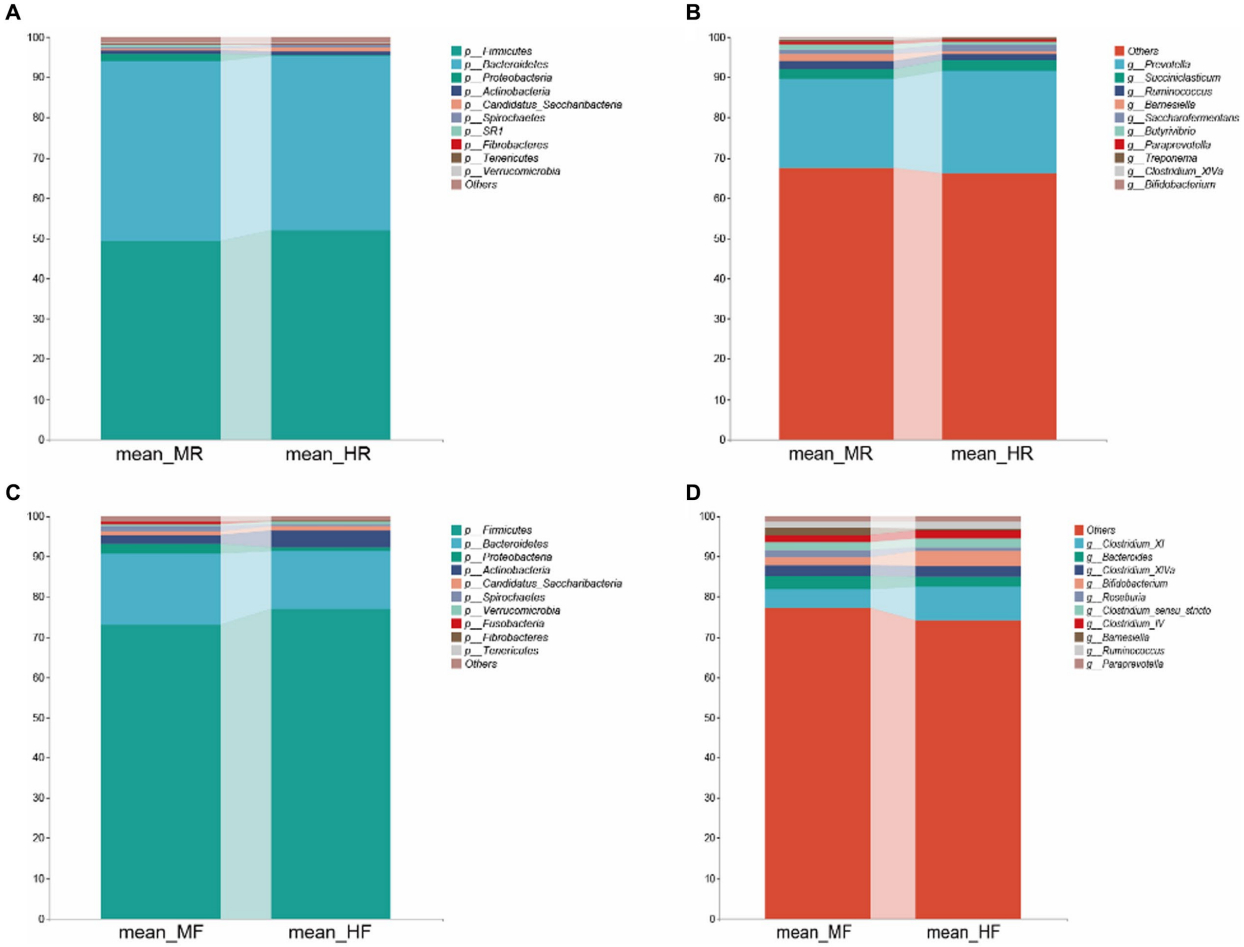
Bacterial composition at the phylum and genus level from the indicated groups. **(A)** and **(C)**: phylum level; **(B)** and **(D)**: genus level; MR, ruminal samples in mastitis group; MF, fecal samples in mastitis group; HR, ruminal samples in health group; HF, fecal samples in health group.

Then, we proceeded with bacterial identification of the samples at different levels. At the phylum level, the top five ruminal bacteria in two groups were *Firmicutes*, *Bacteroidetes*, *Proteobacteria*, *Actinobacteria*, and *Candidatus_Saccharibacteria* (Figure 2A). At genus level, the dominant genera across two groups were *Prevotella*, *Succiniclasticum*, *Ruminococcus*, *Barnesiella*, *Saccharofermentans*, *Butyrivibrio*, *Paraprevotella*, *Treponema*, *Clostridium_XIVa*, and *Bifidobacterium* (Figure 2B). Similarly, at the phylum level of fecal bacteria, the top five microbiota in two group were also *Firmicutes*, *Bacteroidetes*, *Proteobacteria*, *Actinobacteria*, and *Candidatus_Saccharibacteria* (Figure 2C). However, at the genus level, the dominant genera of faces were *Clostridium_XI*, *Bacteroides*, *Clostridium_XlVa*, *Bifidobacterium*, *Roseburia*, *Clostridium_sensu_stricto*, *Clostridium_lV*, *Bamesiella*, *Ruminococcus*, and *Paraprevotella* (Figure 2D). These results indicate that the dominant bacteria in rumen and feces of dairy cows are different.

**Figure 3:**
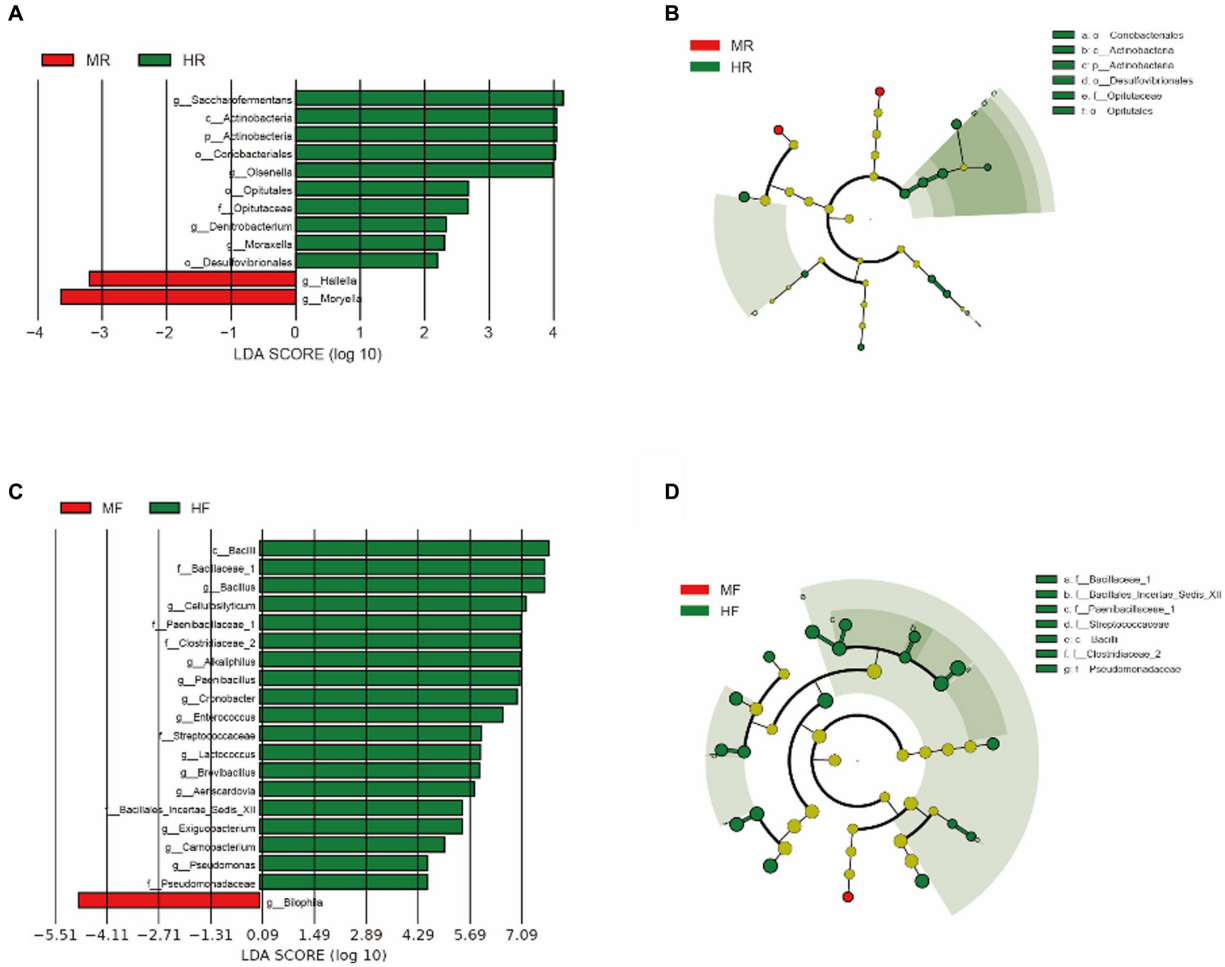
LEfSe analysis was performed to indicate the dominant bacterial taxa enriched in the H and M groups. **(A)** and **(B)** LEfSe results about rumen fluid between H and M group (log_10_LDA score > 2); **(C)** and **(D)** LEfSe results about feces between H and M group (log_10_LDA score > 4); MR, ruminal samples in mastitis group; MF, fecal samples in mastitis group; HR, ruminal samples in health group; HF, fecal samples in health group.

To better understand the dominance of specific bacteria between healthy and mastitis cows, we used the LEfSe method (Figure 3). In the ruminal fluid, we found that the genera *Hallella* and *Moryella* were dominant in mastitis cows, and the genera *Saccharofermentans*, *Olsenella*, *Denitrobacterium*, and *Moraxella* were dominant in healthy cows (Figures 3A,B). In the feces, we only found that the genus *Bilophila* was the dominant in mastits cows while the genera *Bacillus*, *Cellulosilyticum*, *Alkaliphilus*, *Paenibacillus*, *Cronobacter*, *Enterococcus*, *Lactococcus*, *Brevibacillus*, *Aeriscardovia*, *Exiguobacterium*, *Carnobacterium*, and *Pseudomonas* were dominant in healthy cows (Figures 3C,D).

### 3.4 Serum cytokine and LPS correlate with gastrointestinal microbiota

**Figure 4:**
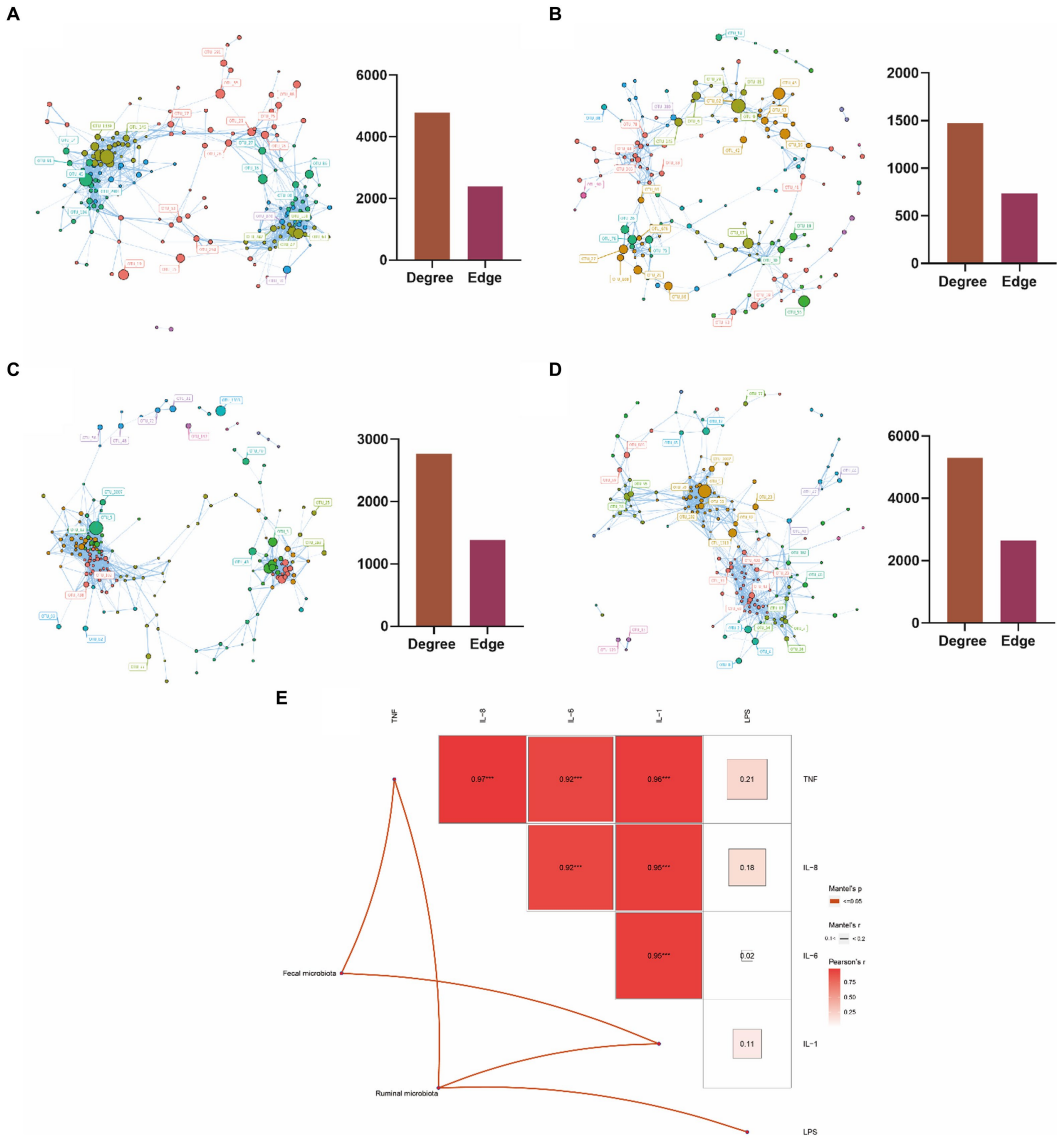
Microbial interaction analysis and the correlation analysis between microbiota and blood metabolic indicators. **(A)** Interaction network analysis of rumen microbiota in mastitis dairy cows; **(B)** interaction network analysis of rumen microbiota in healthy dairy cows; **(C)** interaction network analysis of fecal microbiota in mastitis dairy cows; **(D)** interaction network analysis of fecal micro-biota in healthy dairy cows; **(E)** Mantel analysis of the correlation between ruminal as well as fecal microbiota and blood indicators in dairy cows. Color gradients indicate Pearson’s correlation coefficients. Edge width corresponds to the Mantel’s *r* value (The highest Mantel’s *r* value in this study is 0.1–0.2) and red edge denotes the statistical significance. No edges imply no correlation between microbiota and blood indicators. *0.01 ≤ *p* < 0.05, **0.001 ≤ *p* < 0.01, and ***0.0001 ≤ *p* < 0.001.

To investigate the interaction between the rumen and fecal microbiota in the two groups, we constructed a microbiota interaction network using Spearman correlation. We found that complex interaction networks exist among the microbiota. Interestingly, the rumen microbiota of cows with mastitis exhibited more complex interactions compared to those of healthy cows, with 4,782 vs. 1,474 degrees and 2,392 vs. 738 edges (Figures 4A,B). In contrast, compared with healthy cows, the fecal microbiota of cows with mastitis showed simpler interactions, with 2,766 vs. 5,298 degrees and 1,384 vs. 2,650 edges (Figures 4C,D). Furthermore, we, based on OTUs data, used mantel analysis to explore the relationships between the rumen and fecal microbiota and blood inflammatory and immune indicators in cows. We found that the rumen microbiota was associated with TNF-α, IL-1, and LPS, while the fecal microbiota was associated with TNF-α and IL-1 (Figure 4E). These results reveal that the elevated inflammatory and immune indicators in cows with mastitis are linked to their gastrointestinal microbiota.

### 3.5 PICRUSt2 function prediction

**Figure 5:**
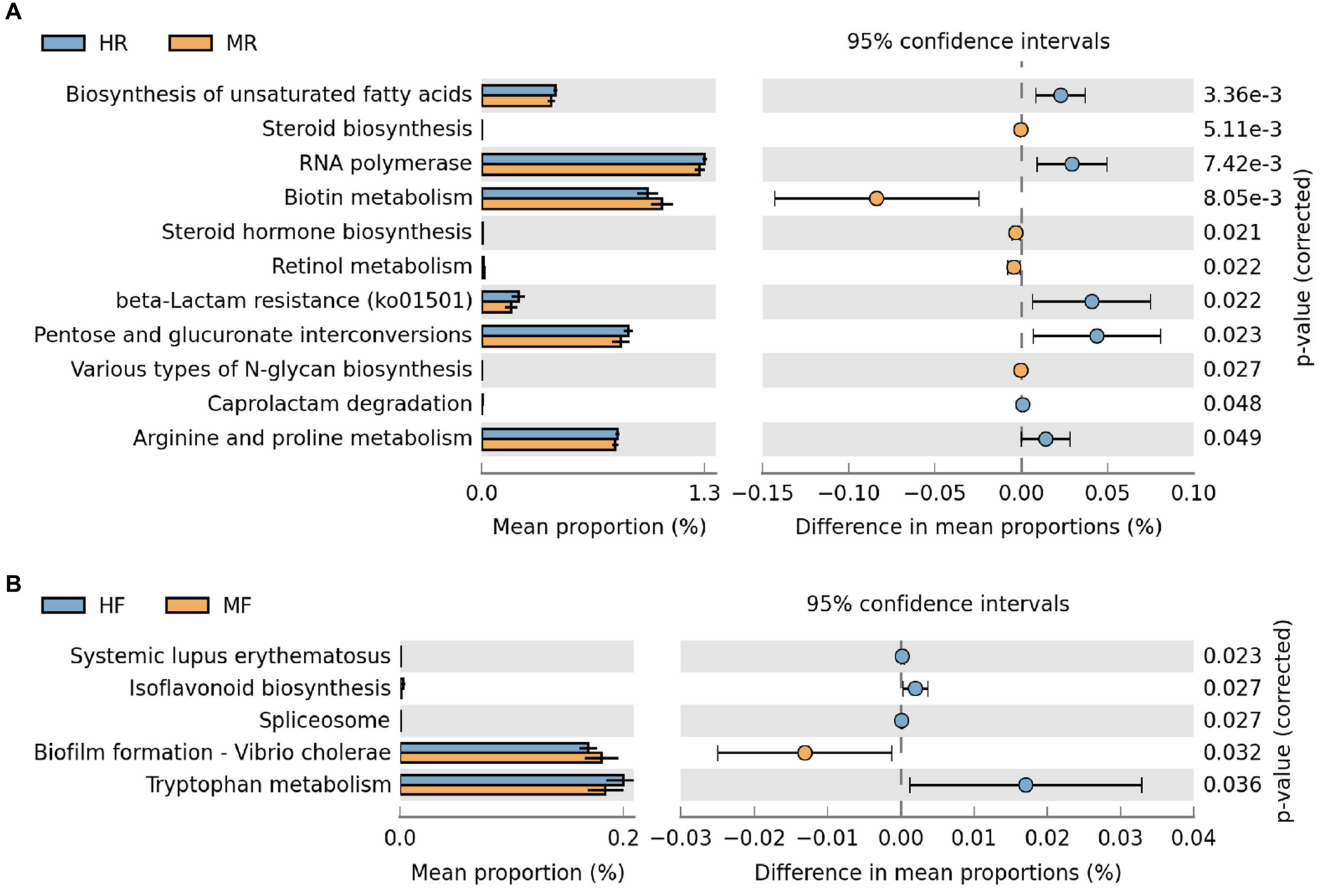
The results of PICRUSt2 function prediction for rumen fluid and feces between the H and M groups (Only showing the significantly different pathways). **(A)** Ruminal function differences were analyzed using STAMP software based on the level 3 pathways. **(B)** Fecal function differences were analyzed using STAMP software based on the level 3 pathways. MR, ruminal samples in mastitis group; MF, fecal samples in mastitis group; HR, ruminal samples in health group; HF, fecal samples in health group.

Furthermore, we conducted statistical analyses on level 3 pathways (Figure 5). In the rumen, pathways such as Steroid Hormone Biosynthesis, Biotin Metabolism, Retinol Metabolism, and Various Types of N-glycan Biosynthesis were found to be significantly upregulated in cows with mastitis. Conversely, in healthy cows, pathways including Biosynthesis of Unsaturated Fatty Acids, RNA Polymerase, Beta-Lactam Resistance, Pentose and Glucuronate Interconversions, Caprolactam Degradation, and Arginine and Proline Metabolism showed significant upregulation (Figure 5A). In fecal samples, pathways such as Systemic Lupus Erythematosus, Isoflavonoid Biosynthesis, Spliceosome, and Tryptophan Metabolism were significantly upregulated in healthy cows. In contrast, the pathway Biofilm Formation – *Vibrio cholerae* was notably upregulated in cows with mastitis (Figure 5B). These findings suggest marked differences in the regulation of metabolic pathways between mastitis-afflicted and healthy cows.

## 4 Discussion

Mastitis is a highly prevalent disease in dairy cows, which causes significant economic losses to the dairy industry. While previous studies have attributed mastitis to the infection of pathogenic bacteria, recent evidence suggests that the gut microbiota of dairy cows may also play a role in the pathogenesis of mastitis (Hu et al., 2019). To the best of our knowledge, this study is the first to investigate the gut microbiota of mastitis and healthy dairy cows while maintaining similar diet, parity, age in days, and lactation period. The results indicate that the gut microbiota of cows with mastitis is different from that of healthy cows, and the differential bacteria may be an important factor contributing to the disease in cows.

Cytokines are commonly used to measure inflammation in the body (Taniguchi and Karin, 2014; Rea et al., 2018). Cows with mastitis exhibit higher concentrations of TNF-α and IL-1 in their blood, consistent with previous studies (Hu et al., 2019; Shangraw and Mcfadden, 2022). This indicates an inflammatory response within the body of cows with mastitis. Similarly, we also observed a significant increase in the levels of LPS in the blood of cows with mastitis. LPS plays a key role in triggering inflammatory responses in the body. When LPS enters the body, its binding with the TLR4 receptor activates downstream signaling pathways such as NF-κB and MAPK (He et al., 2015). The activation of these pathways leads to an increased expression of inflammatory cytokines, such as TNF-α and IL-1 (Luan et al., 2014). Furthermore, LPS are released from the outer membrane of Gram-negative bacteria, containing endotoxin molecules in their lipid components, which are released upon cell death or degradation. Our study’s findings also suggest a correlation between gastrointestinal microbiota and cytokines and LPS. Interestingly, LPS was only associated with the rumen microbiota, and not with the fecal microbiota. This indicates that, in cases of mastitis, the rumen microbiota might play a more significant role than the hindgut. Therefore, the inflammatory response in cows with mastitis might be caused by LPS entering the bloodstream from the rumen. *Moryella* was dominant in the rumen of cows with mastitis and was positively correlated with inflammation indicators. While there is little systematic research on the role of *Moryella* in animal health, studies have shown that the relative abundance of *Moryella* has been found to be associated with obesity in humans (Sung et al., 2017). Additionally, obesity is considered to be a state of chronic low-grade inflammation and is also associated with an increase in inflammatory factors (Saltiel and Olefsky, 2017; Kawai et al., 2021). In addition, *Moryella* is isolated from human pus and is most likely derived from the human gut (Carlier et al., 2007). Therefore, these results also suggest that *Moryella* may contribute to the inflammatory response in cows with mastitis by increasing the production of LPS in the rumen. Interestingly, Zhao et al. found that *Moraxella* and *Saccharofermentans* were the dominant genera in cows with mastitis, whereas our study found that *Moraxella* and *Saccharofermentans* were the dominant genera in healthy cows. Meanwhile, we did not find *Moraxella* and *Saccharofermentans* in the gut microbiota of mice inoculated with rumen fluid from mastitis cows in the validation experiment of microbiota transplantation conducted by Zhao et al. (2022). Therefore, we suggest that *Moraxella* and *Saccharofermentans* may not be the key bacteria causing mastitis, and the divergent results of Zhao et al. may be due to individual cow or external factors. *Olsenella* is a key bacterial genus in healthy dairy cows. Kong et al. (2022) also found that *Olsenella* is positively correlated with the immune and antioxidant capabilities of dairy cows. These results suggest that *Olsenella* may be involved in the regulation of the immune system in dairy cows. Therefore, *Olsenella* in the rumen might make immune systems stronger and help fight mastitis. Although we found no correlation between fecal microbiota and LPS, there is a correlation with TNF-α and IL-1. This suggests that fecal microbiota may have a unique mechanism affecting mastitis. *Bilophila* is a key genus in the feces of cows with mastitis. *Bilophila* often appears in high-fat diets and can worsen metabolic dysfunction in mice. *Bacillus*, *Paenibacillus*, *Brevibacillus*, *Lactococcus*, and *Aeriscardovia* are the dominant genera in healthy cows. *Bacillus* family members have been widely used in ruminants due to their ability to colonize the gut, maintain intestinal homeostasis, and produce beneficial enzymes that enhance dairy cow production (Jeżewska-Frąckowiak et al., 2018; Zhang et al., 2020; Nalla et al., 2022); *Paenibacillus* have nitrogen fixation and antimicrobial properties that could be beneficial for nitrogen utilization and resistance to pathogenic bacteria in dairy cows (Grady et al., 2016); *Aeriscardovia* and *Lactococcus* belong to the Bifidobacterium and Lactobacilli families, respectively, and numerous studies have shown their positive effects on gut health (Sanders et al., 2019; Uusitupa et al., 2020; Widyastuti et al., 2021). These results suggest that the hindgut may maintain cow health by regulating metabolic stability. *Bilophila* might disrupt the hindgut balance in cows, weaken their immunity, and thus cause or worsen mastitis.

Besides the gastrointestinal bacterial composition, we also found differences in microbial interactions between the two groups of cows, which might lead to mastitis. Significant differences in connectivity indicate clear differences in microbial competition and/or cooperation behaviors between the two groups (Dominguez-Bello et al., 2019; Palit et al., 2022). It is well-known that the structure of the rumen in ruminants is unstable, while the hindgut is the opposite. Our study found that the bacterial interactions in cows with mastitis are more complex and structurally more stable. Previous studies have found that a stable structure in the cow’s rumen is a key feature of low cow efficiency (Shabat et al., 2016). Complex microbial interactions can affect cow metabolism, potentially impacting their nutrient needs and leading to reduced immunity (Shabat et al., 2016). The hindgut microbiota plays a crucial role in cow metabolism, and higher stability indicates better tolerance to diseases. This explains why the hindgut microbiota of healthy cows is more robust. Indeed, the results regarding the function of the microbial community also validate the previous discussion. We observed a significant upregulation in the metabolic pathways of unsaturated fatty acids, gluconeogenesis, arginine, and proline in the rumen of healthy dairy cows. Recent research has suggested that glucose can play a role in immune regulation in the body (Ye et al., 2022). Additionally, arginine and proline, as functional amino acids, possess strong immune-regulatory capabilities and are often used as additives to prevent stress in ruminants (Li and Wu, 2018; Wu et al., 2022). This may indicate that healthy dairy cows have better immune-regulatory abilities. Moreover, we observed an upregulation in the synthesis of steroid hormones in the rumen of mastitis dairy cows. Adrenal cortex hormones are known to reduce the body’s absorption of glucose. Therefore, the upregulation of steroid hormones may potentially suppress the immune function of dairy cows.

In summary, we found differences in the gastrointestinal microbiota of healthy and mastitis cows. However, our study did not identify the same dominant genera as other research. We believe that the more comprehensive condition control in our study makes the key genera identified more reliable as a reference. However, further research is needed to verify the causal relationship between these key bacteria and cow health through feeding experiments in germ-free animals. Our next step is to explore the function of microbiome through meta-transcriptomics and combine it with the animal blood or tissue metabolome to better understand the key mechanisms of microbial interactions with the host in mastitis cows.

## 5 Conclusion

This study provides a comprehensive comparison of bacterial composition in the rumen and feces of mastitis and healthy cows, revealing differences in microbiota composition between the two groups. Notably, ruminal *Moryella* may be a key bacteria associated with mastitis, while *Aeriscardovia*, *Lactococcus*, and *Bacillus* in the hindgut of cows may play crucial roles in maintaining cow health. These findings shed new light on mastitis prevention and mechanisms, though further research is needed to verify the identified bacteria and elucidate their interactions with the host.

## Data availability statement

The datasets presented in this study can be found in online repositories. The names of the repository/repositories and accession number(s) can be found at: https://www.ncbi.nlm.nih.gov/, PRJCA000874.

## Ethics statement

The animal study was approved by The Animal Ethics Committee of the China Agricultural University and Jinzhong Vocational and Technical College (Project number: 20210302124338) approved the experimental procedures, and the animal welfare and handling procedures were strictly followed by China Agricultural University experimental guidelines during the experiment. The study was conducted in accordance with the local legislation and institutional requirements.

## Author contributions

CG: Conceptualization, Data curation, Formal analysis, Funding acquisition, Investigation, Methodology, Project administration, Resources, Software, Supervision, Validation, Visualization, Writing – original draft, Writing – review & editing. JL: Data curation, Formal analysis, Methodology, Software, Writing – original draft, Writing – review & editing. YW: Data curation, Methodology, Writing – review & editing. WD: Project administration, Writing – review & editing. SL: Conceptualization, Funding acquisition, Supervision, Writing – review & editing.
